# A Novel 2D Hyperchaotic Map for Secure Financial Data Encryption

**DOI:** 10.3390/e28030262

**Published:** 2026-02-27

**Authors:** Abuduwali Aibai, Mukaidaisi Nuermaimaiti, Yilihamu Tuersun, Dilxat Ghopur

**Affiliations:** 1 School of Finance, Xinjiang University of Finance and Economics, Urumqi 830012, China; abuduwali915@163.com (A.A.); mukaddas16@163.com (M.N.); 2 School of Transportation, Kashi University, Kashi 844006, China; yilihamu26@163.com; 3 School of Computer Science and Technology, Kashi University, Kashi 844006, China

**Keywords:** 2D hyperchaotic map, strong S-Box, AES, encryption algorithm

## Abstract

Given growing concerns regarding data security, we develop an enhanced Advanced Encryption Standard (AES) by incorporating chaotic mapping techniques and implement it within a secure data transmission scheme, thereby strengthening protection mechanisms for both data storage and transmission processes. First, we developed a new 2D enhanced hyperchaotic map (2D-EHM) by combining classical 1D chaotic maps and conducted dynamic testing and analysis using bifurcation diagrams, phase diagrams, Lyapunov exponent graphs, and sample entropy. The results demonstrate that the 2D-EHM exhibits stronger chaotic properties compared to existing chaotic maps. Subsequently, we optimized each step of the AES algorithm by incorporating the proposed chaotic map. The enhanced AES achieves higher security at every stage of the encryption process and utilizes two different strong S-Boxes, effectively addressing the issues related to fixed points, reverse fixed points, and short periodic cycles. Based on this, we designed a secure data transmission scheme. Finally, we conducted a security analysis of the data encryption algorithm, and the results confirm the feasibility and effectiveness of our approach.

## 1. Introduction

In recent years, the rapid advancement of financial technology has transformed the global financial services landscape. From mobile payments and blockchain to high-frequency trading, the scale of financial data transmission and the demand for real-time processing have grown exponentially [1]. The advent of financial technology has revolutionized financial services and forged a new era of convenience and innovation [2]. Digital platforms ranging from mobile banking apps to blockchain-based exchanges have fundamentally reshaped how consumers and businesses manage financial activities, conduct cross-border communications and investments, and have also expanded access to financial services for more social groups [3].

However, this in-depth digital transformation has introduced severe and prominent security challenges: a major concern is the growing vulnerability of financial data to breaches, as the prevalence of online transactions and digital storage of sensitive information has escalated the risk of unauthorized access by cybercriminals, underscoring an urgent need for robust security measures in an increasingly interconnected world [4].

The massive adoption of mobile payments, blockchain technology, high-frequency trading systems, and cross-border settlement platforms has further led to an exponential increase in the volume and frequency of financial data transmission [5]. According to the Bank for International Settlements (BIS), the global daily value of cross-border payments exceeded $6.6 trillion in 2022, with over 70% of these transactions relying on real-time data transmission. Against this backdrop, the confidentiality, integrity, and real-time nature of data have become critical security requirements for financial infrastructure. Meanwhile, the digitalization of the financial system has brought severe security threats: IBM’s “2023 Cost of a Data Breach Report” indicates that the average cost of a single data breach incident in the financial sector reaches $5.97 million, topping all other sectors, with over 40% of breaches attributed to transmission link attacks. Thus, building an efficient, secure, and compliant financial data transmission mechanism has become a core concern for both academia and industry.

AES and RSA, as the most classic symmetric and asymmetric encryption algorithms, have been widely adopted in the field of financial data security [6]. AES is renowned for its high security, efficiency, and scalability, making it a popular choice for safeguarding sensitive data in financial transactions. On the other hand, RSA is typically used for securely exchanging encryption keys, allowing users to share keys over insecure channels, thereby protecting any sensitive information transmitted during a transaction. Both algorithms provide effective data protection suitable for secure data storage and real-time transaction processing [7]. Secure communication channels, digital signatures, and key exchange mechanisms are crucial for verifying identities and safeguarding financial transactions. These mechanisms play a vital role in protecting sensitive financial information across various fintech platforms, including mobile payment applications, online banking portals, and cryptocurrency exchanges.

To further enhance the security and efficiency of financial data encryption, researchers have conducted in-depth research on optimized and hybrid encryption schemes based on traditional algorithms. Kuppuswamy et al. [6] proposed a novel symmetric key algorithm (SSK) and combined it with RSA to construct a hybrid encryption system, which uses RSA for key exchange and SSK for data encryption and decryption to balance security and efficiency in financial communications and transactions. Manna et al. [8] proposed a hybrid cryptosystem combining private and public key models, where the private key itself is encrypted by RSA public key encryption; the scheme is considered to have enhanced security because the shared key intercepted during transmission between senders and receivers is invalid, and it can handle both data transmission and file encryption. Kumar et al. [9] enhanced the AES algorithm for financial data security by increasing the number of encryption rounds to 16 and extending the key length to 320 bits. Tobi et al. [10] introduced the “Image Analysis Encryption Algorithm” to address the challenges of traditional encryption technologies such as RSA and AES in the quantum computing era; this algorithm transforms structured financial data into encrypted images and employs chaotic encryption and fractal analysis to enhance security.

More recently, elliptic curve cryptography (ECC) has emerged as a highly efficient alternative to RSA for key exchange [11]. Its growing popularity in modern cryptographic applications stems from its high efficiency, strong security, and resilience against various attacks, which is primarily due to the inherent difficulty of solving the elliptic curve discrete logarithm problem (ECDLP). With significantly smaller key sizes than RSA, ECC is particularly suitable for resource-constrained environments such as mobile payment applications, which are critical in fintech scenarios. The widespread application potential of ECC is further evidenced by related research: Adhikari et al. [12] introduced a PRNG based on large prime ECs, which extracts the least significant 8 bits of the y-coordinate from generated curve points to produce pseudo-random numbers; Hayat et al. [13] proposed another EC-based PRNG, though their approach of generating and sorting all curve points leads to high computational costs.

In addition, chaotic maps have emerged as a compelling and promising area of research in the field of cryptography (Feng et al. [14]). They are characterized by intricate dynamics, including remarkable sensitivity to initial conditions, non-linearity, and strong randomness, and these distinctive properties make chaotic maps particularly effective for cryptographic applications, prompting researchers to explore their various applications in data security [15,16]. For example, Podder et al. [17] proposed a financial security encryption algorithm based on the Logistic map, which incorporates a dual confusion process followed by a diffusion process: a divide-rotate algorithm is employed in the first confusion phase, a pixel shifting algorithm in the second, and a pseudo-random sequence generator is used to produce chaotic values for image diffusion. Kadeer [18] introduced a 2D hyperchaotic map with strong chaotic performance, which was further utilized to develop an S-Box construction algorithm and a word-wise stream cipher for enhancing data security. Yu et al. [19] further explored the application of chaotic systems in information security by proposing a multiscroll Hopfield Neural Network based on a non-polynomial memristor; they realized its hardware implementation via FPGA and designed a dedicated image encryption circuit, which provides a valuable hardware reference for the engineering application of chaotic systems in cryptographic scenarios.

However, most existing algorithms, including AES, still exhibit critical shortcomings. For instance, encryption schemes employing S-Boxes often suffer from weak S-Box designs characterized by fixed points (e.g., S(x)=x), reverse fixed points (e.g., $S(x)=\bar{x}$), and short periodic cycles, significantly undermining cryptographic security. Symmetric encryption algorithms face inherent challenges in secure key exchange mechanisms. While RSA remains viable for key exchange, its reliance on excessively long keys results in prohibitive computational overhead, rendering encryption speeds three orders of magnitude slower than symmetric counterparts—thus limiting its utility primarily to key negotiation. Furthermore, most chaos-based cryptographic systems adopt 1D chaotic maps (e.g., logistic, sine, and quadratic maps), which exhibit insufficient randomness, non-ergodic behavior, and limited chaotic range. Across most parameter ranges, these maps fail to demonstrate sensitivity to initial conditions, a hallmark of robust chaotic dynamics.

To address the aforementioned critical issues in current financial data encryption, this paper conducts targeted research and proposes a secure financial data encryption and transmission scheme based on an optimized AES algorithm combined with a self-constructed chaotic map. Specifically, we first construct a novel 2D hyperchaotic map (2D-EHM) and conduct in-depth dynamic analysis, which verifies that the map exhibits strong and stable chaotic behavior over a wide parameter range. On this basis, we develop two high-performance robust S-Boxes that feature high non-linearity and are free of defects such as fixed points, reverse fixed points, and short periodic cycles. We then optimize the classic AES algorithm by integrating the 2D-EHM and the two proposed S-Boxes into its core encryption process, and finally design a complete method for encrypting and transmitting financial data based on this enhanced AES algorithm.

The integration of chaotic maps into the AES framework enhances the cryptographic security of the algorithm in a more systematic way than merely introducing randomness. Traditional AES, while robust in general, relies on fixed components such as a static S-Box with inherent weaknesses and a deterministic key schedule, which can be vulnerable to sophisticated cryptanalysis if not properly implemented. Our optimized approach leverages the inherent excellent properties of the 2D-EHM to address these potential drawbacks: specifically, the extreme initial-value sensitivity of the 2D-EHM ensures that a minute change in the encryption key results in a drastically different and unpredictable ciphertext, which significantly strengthens the algorithm’s resistance against differential attacks. Furthermore, the ergodicity and high nonlinearity of the 2D-EHM allow us to dynamically optimize the core cryptographic components of AES, effectively eliminating the security risks associated with fixed S-Boxes such as fixed points, reverse fixed points, and short iterating cycles. In essence, the 2D-EHM provides a dynamic and complex source of nonlinearity that systematically fortifies each stage of the AES encryption process, creating a more resilient and unpredictable security architecture that is more adapted to the high security requirements of financial data.

The structure of the paper is organized as follows: Section 2 analyzes chaotic maps and introduces the design and dynamic characteristics of the 2D-EHM. Section 3 proposes two robust S-Boxes and verifies their performance. Section 4 outlines the detailed design of the chaotic encryption algorithm based on the optimized AES. Section 5 describes the design of a secure and efficient financial data transmission scheme based on the proposed encryption algorithm. Section 6 presents a comprehensive performance and security analysis of the proposed methods through experimental verification. Finally, Section 7 concludes the full paper and discusses potential directions for future research.

## 2. Chaotic Maps and Analysis

### 2.1. Classical Logistic and Quadratic Map

All numerical simulations and analyses of the logistic and quadratic chaotic maps were performed using MATLAB R2018b, executed on a workstation equipped with a 12th Gen Intel^®^ Core™ i7-12650H (2.30 GHz) processor.

In the study of chaotic systems, the logistic map and the quadratic map are among the most commonly used chaotic maps, garnering significant attention and application across various fields. These two maps not only hold theoretical importance but also play crucial roles in biology, economics, physics, and many other domains.

The mathematical expressions for the logistic map and the quadratic map are given by Equations (1) and (2), respectively:

(1) $$x_{n+1}=rx_{n}(1-x_{n})$$

(2) $$x_{n+1}=r-{x_{n}}^{2}$$

where $x_{n}$ is the state variable and r is a control parameter.

**Figure 1:**
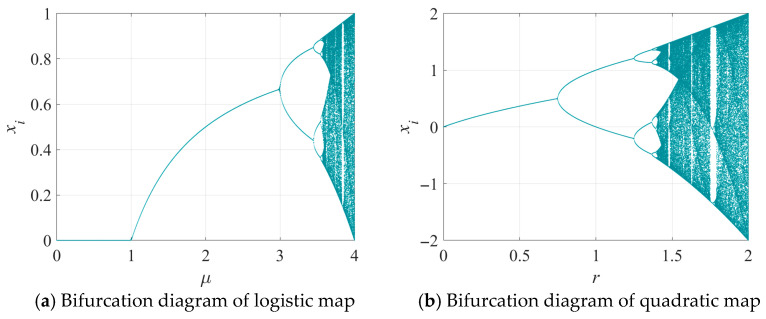
Bifurcation diagrams of classical chaotic maps: (**a**) Logistic map; (**b**) Quadratic map.

**Figure 2:**
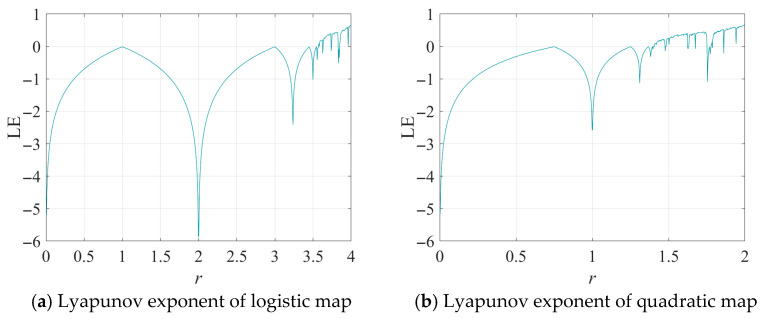
Lyapunov exponents of classical chaotic maps: (**a**) Logistic map; (**b**) Quadratic map.

As shown in Figure 1 and Figure 2, both maps exhibit non-chaotic behavior in most regions, with several blank windows indicating a lack of ergodicity. Additionally, the Lyapunov exponent is found to be less than 0 in the majority of these regions, indicating a low level of randomness and demonstrating stability and predictability.

### 2.2. 2D-EHM

To overcome the limitations of 1D chaotic mappings, the 2D-EHM is constructed by combining the logistic map and the quadratic map. It is defined as Equation (3):

(3) $$\left\{\begin{array}{l} x_{i+1}=\lambda r(x_{i}-{y_{i}}^{2})\mathrm{mod}1\\ y_{i+1}=\theta y_{i}(r-{x_{i}}^{2})\mathrm{mod}1 \end{array}\right.$$

where (x,y)∈[0,1) represent the state variables, r is the global control parameter, and λ and θ are dimensional gain parameters that regulate the nonlinear evolution intensity and chaotic behavior of the *x*-dimensional and *y*-dimensional components, respectively.

**Figure 3:**
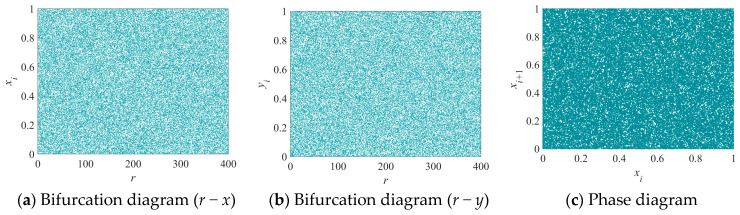
Bifurcation and phase diagrams of the 2D-EHM: (**a**) *r* − *x* bifurcation diagram; (**b**) *r* − *y* bifurcation diagram; (**c**) Phase diagram.

As shown in Figure 3, the 2D-EHM exhibits chaotic behavior over a wide range and demonstrates ergodicity. This indicates that the system not only fills the phase space effectively but also ensures that its trajectory will eventually explore all accessible states, making it suitable for various applications that require robust chaotic characteristics.

### 2.3. Lyapunov Exponent and Sample Entropy

The Lyapunov exponent, as one of the most important metrics of chaotic maps, serves to evaluate the strength of chaotic characteristics. A Lyapunov exponent greater than 0 indicates that the system has entered a chaotic state. A Lyapunov exponent equal to 0 implies that the system is in a neutral state. Conversely, a Lyapunov exponent less than 0 signifies stability, suggesting that the system returns to a stable equilibrium after small disturbances, reflecting predictable behavior.

Sample entropy provides a numerical assessment of the complexity and irregularity present in a sequence. A higher sample entropy value indicates a higher level of complexity and greater irregularity within the sequence, reflecting its intricate and unpredictable nature.

**Figure 4:**
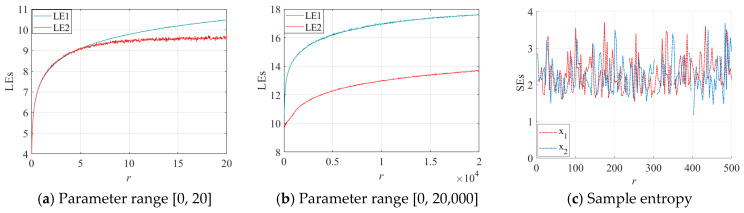
Lyapunov exponents and sample entropy of the 2D-EHM: (**a**) LEs with a parameter range of [0, 20]; (**b**) LEs with a parameter range of [0, 20,000]; (**c**) Sample entropy.

As shown in Figure 4a,b, both Lyapunov exponents of the 2D-EHM are greater than 0, indicating that it is a hyperchaotic map. Figure 4c demonstrates that the sample entropy values range between 2 and 3, indicating a strong level of complexity and irregularity. These two metrics together suggest that the 2D-EHM has promising applications in the field of data encryption, particularly in the encryption of financial data. Such characteristics enhance the confidentiality and resilience of the data, providing reliable support for the protection of financial information.

## 3. S-Box Construction and Comparison

As a core component of the proposed data encryption algorithm, the S-Box directly determines the nonlinearity and anti-attack capability of the entire encryption system. To overcome the security defects of fixed S-boxes in traditional AES and construct a strong S-Box with high nonlinearity, this chapter utilizes the S-Box construction and optimization method described in our previous paper [20]. This method effectively eliminates common drawbacks of conventional S-boxes, including fixed points, reverse fixed points, and short periodic cycles, thereby improving the overall security of the S-Box and laying a solid foundation for the dynamic enhanced AES algorithm.

Through the aforementioned construction and optimization method, two robust 8 × 8 S-Boxes (denoted as S1 and S2) are constructed. These two S-Boxes are designed to be compatible with the dynamic enhancement mechanism of the proposed algorithm—they can be dynamically adjusted during the SubBytes step of AES, breaking the static limitation of traditional AES S-boxes and enhancing the algorithm’s resistance to differential and linear cryptanalysis. Their strict bijection and high nonlinearity further lay a solid foundation for improving the overall security of the encryption system.

As shown in Table 1, both S1 and S2 are 8 × 8 S-Boxes with strict bijection, a fundamental requirement for AES SubBytes reversibility. As verified by Table 2 and Table 3, each byte (00~FF) appears exactly once in both S-Boxes, ensuring no data loss during encryption and decryption.

In terms of security performance, combined with Table 1, S1 and S2 have high nonlinearity (min 110, max 112), slightly lower than AES S-Box but significantly higher than Whirlpool S-Box, ZUC S0 and other mainstream S-Boxes, effectively resisting linear and differential cryptanalysis. Their average SAC values are close to the ideal 0.5, exhibiting good avalanche effects and enhancing input-output correlation resistance.

A key advantage is that S_1_ and S_2_ eliminate fixed points, reverse fixed points and short iterating cycles—weaknesses existing in AES, Whirlpool and ZUC S-Boxes—making them more secure for financial data encryption.

In summary, the two 8 × 8 S-Boxes S1 and S2 constructed in this chapter have excellent structural characteristics and security performance, including strict bijection, high nonlinearity, good SAC performance, and no fixed points, reverse fixed points, or short periodic cycles. These superior characteristics enable the S-Boxes to effectively address the security defects of fixed S-Boxes in traditional AES, and they can be dynamically selected and applied in the SubBytes step of the proposed dynamic enhanced AES financial data encryption algorithm, thereby further improving the overall security and anti-attack capability of the encryption system, and providing strong support for the secure transmission and storage of financial data.

## 4. Design of the Chaotic Encryption Algorithm

This section proposes a symmetric encryption algorithm based on the constructed 2D-EHM. The core idea is to generate a dynamic key stream through the pseudorandomness of the chaotic system, combined with confusion and diffusion operations to achieve the conversion from plaintext to ciphertext. The algorithm supports variable-length keys (ranging from 128 to 256 bits) and is suitable for encrypting structured financial data such as text and transaction messages.

### 4.1. Preparatory Work

Step 1: key generation

(1) Combine the input initial key (*K*) with its length *L* = length(*K*), and then calculate its hash value using Equation (4).

(4) H=hash(K||L)

(2) Generate the initial value (*x*_0_, *y*_0_) and parameters using Equation (5).

(5) $$\left\{\begin{array}{l} x_{0}=(\sqrt{5}\mathrm{sum}(H(1:20)))\mathrm{mod}1\\ y_{0}=(\sqrt{13}\mathrm{sum}(H(11:30)))\mathrm{mod}1\\ r=15+\frac{17}{\sqrt{3}}(\mathrm{sum}(H(21:40))\mathrm{mod}1\\ \lambda =7\times 10^{3}+(\mathrm{sum}(H(31:50))\mathrm{mod}25\\ \theta =2\times 10^{2}+(\mathrm{sum}(H(41:64))\mathrm{mod}25 \end{array}\right.$$

Step 2: Plaintext grouping

(1) Divide the plaintext into blocks, each with a length of 256 bits (32 bytes). If the length of the plaintext is not a multiple of 32 bytes, it is padded with zeros.

(2) Consider each block as an 8×4 matrix, and fill it in column-major order.

$$\begin{array}{c} P_{i}=\left[\begin{array}{llll} b_{0} & b_{8} & b_{16} & b_{24}\\ b_{1} & b_{9} & b_{17} & b_{25}\\ b_{2} & b_{10} & b_{18} & b_{26}\\ b_{3} & b_{11} & b_{19} & b_{27}\\ b_{4} & b_{12} & b_{20} & b_{28}\\ b_{5} & b_{13} & b_{21} & b_{29}\\ b_{6} & b_{14} & b_{22} & b_{30}\\ b_{7} & b_{15} & b_{23} & b_{31} \end{array}\right] \end{array},\;i=1,2,\ldots ,n.$$

### 4.2. Round Parameter Generation

Step 1: chaotic sequence generation

(1) Iterate the 2D-EHM 96 times to obtain chaotic sequence.

Step 2: Diffusion matrix generation

(2) Fill the first 64 integers into an 8×8 matrix $M_{r}$ in column-major order using Equation (6).

(6) $$M_{r}(i,j)=(\pi^{x_{l}}+e^{y_{l}})\mathrm{mod}256$$

where 0≤i,j<8 and l=1,2,…,64.

(3) Calculate the determinant of the matrix $M_{r}$ using Equation (7). If it is not invertible, jump to Step 1 to re-iterate the chaotic map and regenerate the chaotic sequence.

(7) $$\left|M_{r}\right|=\sum_{j=1}^{n}a_{ij}A_{ij}=a_{i1}A_{i1}+a_{i2}A_{i2}+\cdots +a_{in}A_{in}$$

where the algebraic cofactor is $A_{ij}={\left(-1\right)}^{i+j}M_{ij}$, where $M_{ij}$ denotes the determinant of the (*n* − 1)-order minor matrix obtained by deleting the *i*-th row and *j*-th column of $M_{r}$.

Step 2: Round key expansion

Obtain a round key ${key}_{r}$ using Equation (8).

(8) $$key_{r}(i)=(\pi^{x_{l}}+e^{y_{l}})\mathrm{mod}256$$

where 0≤i<32 and l=65,66,…,96.

### 4.3. Encryption Process

Our encryption algorithm has a dynamic number of encryption rounds, specifically performing *d* rounds of encryption, where d=5+length(K)mod4.

Round 1~(d−1):

Step 1: SubBytes

(1) Odd Rounds: Perform byte substitution using the first S-Box S_1_ using Equation (9).

(9) $$b_{i,j}^{\prime }=S_{1}(b_{i,j})$$

(2) Even Rounds: Perform byte substitution using the second S-Box S_2_ using Equation (10).

(10) $$b_{i,j}^{\prime }=S_{2}(b_{i,j})$$

Step 2: ShiftRows

Perform a cyclic left shift on each row by m=1+tmod3 bits, where *t* represents the encryption round number.

Step 3: MixColumns

Perform matrix multiplication on each column ${col}_{j}$ using Equation (11).

(11) $$col_{j}^{\prime }=col_{j}\mathrm{mod}256$$

where ${col}_{j}$ is the columns after step 2.

Step 4: AddRoundKey

Perform a byte-wise XOR operation between the round key ${key}_{r}$ and the matrix using Equation (12).

(12) $$b_{i,j}^{\prime }=b_{i,j}⊕key_{r}(4i+j)$$

Round *d* (final round) skips Step 3.

**Figure 5:**
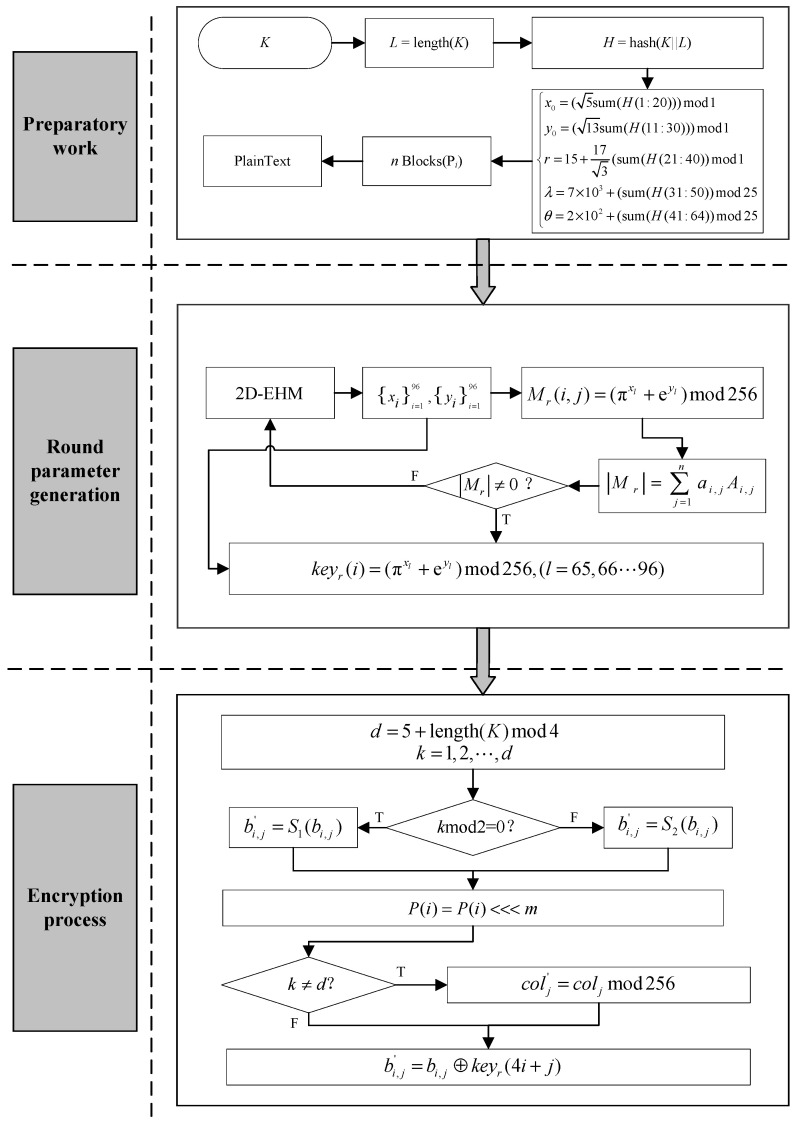
Flowchart of the proposed encryption algorithm.

Figure 5 presents the flowchart of the proposed encryption algorithm.

### 4.4. Decryption Process

Perform the encryption steps in reverse order, using the inverse of the S-Boxes and the inverse of the matrix for column diffusion.

## 5. Design of Secure Financial Data Transmission Scheme

This section integrates the proposed chaotic encryption algorithms with financial data transmission scenarios to design an end-to-end secure protocol. The transmission scenarios include real-time payments such as cross-border remittances, securities transactions such as stock orders, and data sharing such as credit report inquiries.

### 5.1. Transmission Structure

1. Layered architecture design

Application layer: Business systems generate structured financial data.

Security layer: Integrate the proposed chaotic encryption algorithms for real-time business data encryption.

Transmission layer: Build secure channel based on TLS 1.3 protocol, compatible with SWIFT/CFETS and other financial communication protocols.

Key management layer: Financial institution internal KMS, supporting initial key rotation and secure storage.

### 5.2. Secure Transmission Solution

1. Participating roles

Data sender: Bank, brokerage, payment institution terminals

Data receiver: Clearing center, exchange, third-party financial institutions

Regulator: Central bank, financial regulatory agencies

2. Data transmission

Step 1: The client splits structured financial plaintext into 128-bit blocks and pads to 16 bytes.

Step 2: Generate a round key based on initial key K and perform 5 rounds of confusion encryption.

Step 3: Encapsulate the ciphertext *C* and timestamp *t_s_*, and transmit it through TLS.

**Figure 6:**
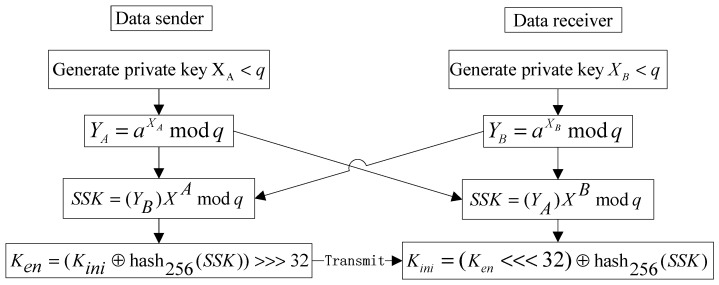
Flowchart of the key transmission.

Step 4: Realize secure key transmission from sender to receiver based on Diffie–Hellman protocol. Figure 6 presents a detailed flowchart of the key transmission process.

3. Decrypt verification

Server verifies timestamp validity (valid time window: ± 2 s) to resist replay attacks.

Use the key obtained by Diffie–Hellman protocol to perform reverse decryption of ciphertext.

Check decrypted financial data integrity; terminate transmission and alarm if tampering is found.

### 5.3. Experimental Verification

We select two representative types of financial transaction data, and standardize all data by concatenating core business fields in their native logical order and then formatting them into 128-bit fixed-length blocks. The specific data structure and format are shown in Table 4.

We use the standardized 128-bit hexadecimal data blocks from Table 4 as the original plaintext. We first divide the financial data into fixed-length blocks, then encrypt and decrypt them in strict accordance with the steps outlined in Section 5.2. Finally, we conduct an analysis on key metrics.

To intuitively demonstrate the encryption effect on financial data, we select typical samples of the two data types. Table 5 presents the comparison between the 128-bit plaintext and the encrypted ciphertext.

The experimental results based on financial transaction datasets demonstrate that our proposed secure transmission scheme can efficiently encrypt financial data blocks. The encrypted ciphertext completely conceals the business characteristics of the original data, and the decrypted data is identical to the plaintext.

## 6. Performance and Security Analysis

This section outlines the empirical research conducted to assess the performance of the proposed data encryption algorithm.

### 6.1. Key Space

The size of the key space directly affects the security of encryption systems. A larger key space means that an attacker needs to try more key combinations to successfully crack the encryption, thereby enhancing data security [23]. To effectively resist brute-force attacks, an ideal key space should be greater than 2^128^. To ensure the strength and security of the encryption system, our algorithm design features a key space corresponding to a 256-bit hash value, equivalent to 2^128^. Considering that the initial values can be precise to 10^−15^, we set the initial conditions for 2D-NDQM as $\left(x_{0},y_{0},r,\lambda ,\theta \right)$, which can be expanded to $5\times 10^{15}$. Therefore, the overall key space expands to $2^{128}\times 5\times 10^{15}>2^{180}$, providing ample size to withstand common cryptographic attacks. This design not only enhances the security of the system but also offers greater protection for future applications.

### 6.2. Key Sensitivity

Key sensitivity refers to the degree to which an encryption algorithm is sensitive to changes in the key, meaning that small changes in the key will result in significant changes in the encryption output. A good encryption algorithm should possess high key sensitivity, which implies that even if a single bit of the key is altered, the resulting ciphertext should exhibit substantial variation. This characteristic can effectively prevent attackers from inferring the content of the key through ciphertext analysis.

NBCR (Number of Bit Change Rate) is an indicator used to assess the key sensitivity of encryption algorithms. It measures the ratio of the number of bit changes in the ciphertext resulting from minor changes in the key to the total number of bits in the ciphertext. A high NBCR value indicates that the algorithm is sensitive to key changes and has good security, while a low NBCR value may suggest that the algorithm is not sensitive to key changes, thereby reducing its security.

To analyze the key sensitivity of the proposed encryption algorithm, we first selected the initial key ‘password0’ and then generated five additional keys by changing the last ‘0’ to each of the digits from ‘0’ to ‘5’. We then used these six keys to encrypt two plaintexts (with lengths of 10,000 bits and 20,000 bits, respectively). Finally, we compared the number of differing bits between the ciphertexts and calculated the NBCR values. As shown in Table 6, the NBCR values are close to the ideal values, indicating that the proposed encryption algorithm has strong key sensitivity.

### 6.3. Hamming Distance

Hamming distance serves as a key metric in evaluating the security of encryption algorithms. It quantifies how much the output changes in response to minor modifications in the input, illustrating the concept of the avalanche effect. In an ideal scenario, the Hamming Distance should approach 50%, indicating that even a small tweak in the encryption key should lead to a substantial transformation in the resulting ciphertext [24]. This property is crucial for ensuring that encrypted data remains secure and resistant to potential attacks.

**Figure 7:**
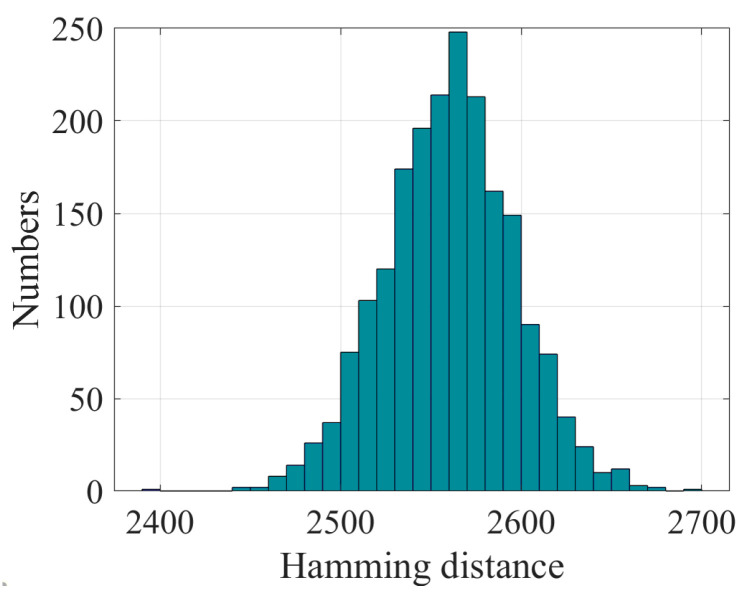
Hamming distance distribution.

We encrypted a plaintext of length 5120 bits using slightly different keys and calculated the Hamming distance between them. As shown in Figure 7, the results are very close to the ideal value.

### 6.4. Correlation Evaluation

Correlation evaluation is a statistical method used to analyze the relationship between two sets of data, particularly in the context of cryptography. In encryption, it helps to determine whether there is any correlation between the plaintext and the ciphertext. A strong encryption algorithm should ideally produce ciphertext that is statistically independent of the plaintext, meaning that changes in the plaintext do not lead to predictable changes in the ciphertext. This independence is crucial for ensuring the security of the encryption scheme against various types of attacks. The correlation coefficient is a numerical measure that quantifies the degree to which two variables are related. It ranges from −1 to 1, where −1 indicates a perfect negative correlation, 1 indicates a perfect positive correlation, and 0 indicates no correlation at all. In cryptographic contexts, a correlation coefficient close to 0 suggests that the ciphertext does not reveal any information about the plaintext, which is a desirable property for secure encryption.

**Figure 8:**
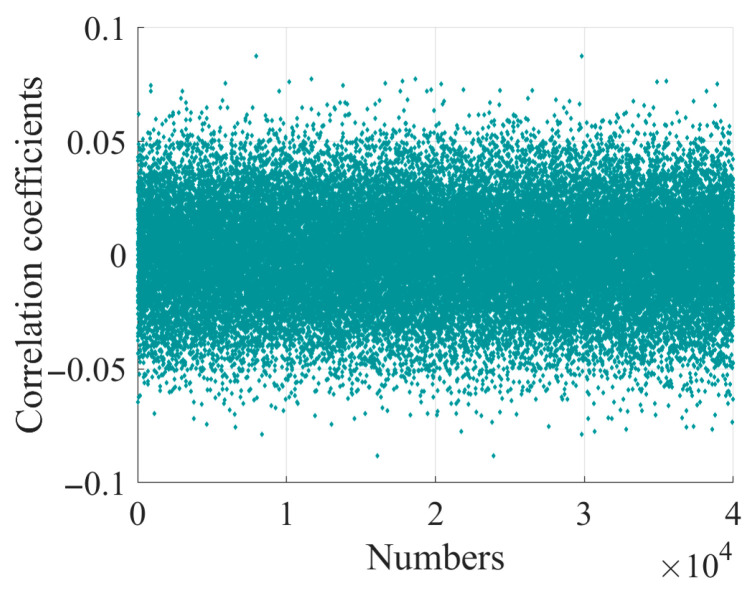
Correlation coefficients distribution.

To evaluate the correlation, we encrypted a 16,000-bit plaintext 300 times and calculated the correlation coefficients. As shown in Figure 8, the correlation coefficients are concentrated between −0.04 and 0.04, with an average value of 0.000611. This indicates that there is minimal correlation between the plaintext and the ciphertext, reinforcing the effectiveness of the encryption algorithm.

### 6.5. Randomness Testing

Randomness is crucial in encryption algorithms because it impacts the security and effectiveness of the entire encryption process. High randomness ensures that encryption algorithms can produce unpredictable outputs, which is essential for preventing attackers from inferring information by analyzing encrypted data. Without sufficient randomness, encryption algorithms may become vulnerable and susceptible to various attacks.

In this context, TestU01 is a comprehensive software suite specifically designed for the rigorous evaluation of binary sequences, covering algorithms such as Rabbit, Alphabit, and BlockAlphabit. We conducted 38 and 17 different statistical tests on Rabbit and Alphabit, respectively, using TestU01 1.2.3 to assess the randomness of the binary sequences they generate. Additionally, BlockAlphabit evaluates the sequences by reordering bits into different block sizes (2, 4, 8, 16, and 32 bits) and repeatedly applying the tests from Alphabit. We tested binary sequences of lengths $L=2^{15}$ and $L=2^{25}$, and the results, as shown in Table 7, indicate that all tests were successfully passed. This demonstrates that the binary sequences generated by these algorithms possess good randomness and are suitable for applications with high security requirements.

### 6.6. Advantages of Our Work

1. 2D hyperchaotic map with enhanced properties

We designed a novel 2D hyperchaotic map, 2D-EHM, by combining the classic logistic map and the quadratic map. This map exhibits the following advantages:

(1) Wider chaotic range: Demonstrates hyperchaotic behavior across all of the parameter space, significantly broader than most of the existing chaotic maps.
(2) High sensitivity: A minor perturbation of the initial conditions triggers completely divergent trajectories, ensuring unpredictability.
(3) Ergodicity: The designed 2D hyperchaotic map exhibits strong ergodic properties, meaning that its trajectories densely cover the entire phase space over time. This characteristic ensures that, given enough time, the system can reach any point in the state space, making it highly suitable for applications in financial data encryption.

2. Dual strong S-Boxes

The proposed algorithm employs two strong S-Boxes that are alternately applied in odd and even encryption rounds, significantly enhancing the overall security of the encryption process. Both S-Boxes exhibit a nonlinearity of over 111, which is significantly higher than that of most existing S-Boxes and contributes to their strength against linear cryptanalysis. Furthermore, they are designed without fixed points, reverse fixed points, or short periodic cycles, eliminating potential vulnerabilities that could be exploited by attackers. This dual S-Box structure not only increases the complexity of the encryption but also ensures a higher level of resistance against various attacks.

3. Key strengthening

To address the issue of weak keys, such as all-zero or all-one keys, a clever method has been proposed that combines the key with its length and computes the hash value of the key. Subsequently, a nonlinear equation is used to further process the hash value to generate the initial key. This method not only enhances the security of the key but also effectively mitigates the security risks associated with weak keys, thereby improving the robustness and attack resistance of the overall encryption algorithm.

4. AES optimization with hyperchaotic map

Optimize all steps of AES using the proposed hyperchaotic map. Implement dynamic use of two strong S-Boxes, and in addition, each round of encryption utilizes a dynamically generated column mixing matrix and round key.

5. Secure transmission scheme for financial data:

Finally, based on the proposed encryption algorithm, a financial data encryption transmission scheme is designed. This scheme fully leverages the advantages of the hyperchaotic map. In addition, the Diffie–Hellman key exchange protocol is used to securely transmit the key from the data sender to the data receiver.

Table 8 presents a brief comparison between our work and other encryption algorithms.

## 7. Conclusions

In the context of the rapidly evolving information age, the demand for financial data security has become increasingly critical. As financial transactions and data exchanges grow more prevalent in digital formats, ensuring the confidentiality and integrity of this information is paramount. In order to address these challenges, we proposed an effective encryption scheme for financial data transmission.

We initially developed a 2D hyperchaotic map, 2D-EHM, characterized by robust chaotic properties and conducted a dynamic analysis. This chaotic map exhibits a high level of randomness and unpredictability, along with superior ergodicity compared to most existing chaotic maps. Subsequently, we designed two strong S-Boxes that avoid weaknesses such as fixed points, reverse fixed points, and short periodic cycles. We then optimized AES using the chaotic map and the two strong S-Boxes, achieving dynamic operations at each step. Finally, we designed a financial data encryption transmission scheme, and the test results validated its effectiveness and security.

## Figures and Tables

**Table 1:** Performance comparison of S-Boxes.

	Non-Linearity			SAC							
S-Box	Min	Max	Avg	Min	Max	Avg	BIC_SAC	BIC_NL	DAP	LAP	Weaknesses
Table 1	110	112	111.5	0.4531	0.5625	0.5061	0.5048	111.429	0.0234	0.0859	None
Table 2	110	112	111.25	0.4375	0.5937	0.5041	0.5053	110.571	0.0234	0.0859	None
S-Box in AES	112	112	112	0.4531	0.5625	0.5049	0.5046	112	0.0156	0.0625	5 short cycles
S-Box in Whirlpool	110	108	104.5	0.4062	0.6094	0.5149	0.5069	104.071	0.0313	0.1094	2 reverse fixed points and 12 short cycles
S0 in ZUC	96	104	98	0.3438	0.625	0.4948	0.4951	101.714	0.0312	0.1250	reverse fixed point and 2 short cycles
S1 in ZUC	112	112	112	0.4375	0.5625	0.5093	0.5059	112	0.0156	0.0625	5 short cycles
Ref. [21]	106	110	107.5	0.4375	0.5781	0.5097	0.5068	103.92	0.0391	0.1250	fixed points and 7 short cycles
Ref. [22]	103	107	105.375	0.4140	0.5703	0.5012	0.4978	103.821	0.0468	0.1445	2 reverse fixed points and 7 short cycles

**Table 2:** The first strong S-Box S_1_.

	0	1	2	3	4	5	6	7	8	9	A	B	C	D	E	F
0	30	C6	CB	C8	D1	48	4A	1F	E5	2D	4B	68	D2	9B	78	C3
1	49	31	1C	CE	D0	8C	0B	D5	7E	97	71	7A	B6	77	C1	15
2	FB	DB	B9	63	E3	EA	DE	16	E7	7F	5F	DD	CD	94	ED	AF
3	27	1B	69	19	A4	B3	2F	B0	2B	A1	35	51	58	6B	F1	CF
4	2C	39	66	00	A8	42	80	75	81	E8	93	F9	6C	59	6A	37
5	89	A0	25	5E	65	D6	FD	88	40	18	F2	EC	01	06	84	1A
6	95	5A	70	D8	09	0E	E9	3F	0F	DC	21	CA	85	E6	BA	74
7	8D	79	05	3A	B1	BE	E4	DF	F6	F3	90	6D	A5	DA	D9	91
8	1E	26	A9	56	8A	BB	07	AB	17	7B	C2	EE	47	8E	AC	C9
9	45	3D	0A	96	60	61	B5	34	03	52	F4	A7	92	82	28	98
A	55	E1	E0	20	0C	23	67	86	11	99	76	41	BD	BF	57	CC
B	5B	24	EB	4E	3E	9F	02	7C	46	83	D7	50	4F	C0	72	9D
C	F0	C4	6F	62	A6	73	F7	13	54	9E	C7	AA	08	FE	38	14
D	C5	E2	FF	43	04	29	D3	22	4D	EF	8B	FC	33	1D	AE	B2
E	5D	D4	B4	AD	4C	9C	32	B7	B8	A2	3B	5C	12	8F	64	9A
F	36	7D	3C	2E	FA	53	10	44	0D	BC	6E	2A	F5	87	F8	A3

**Table 3:** The second strong S-Box S_2_.

	0	1	2	3	4	5	6	7	8	9	A	B	C	D	E	F
0	75	FA	F7	F4	ED	74	76	23	D9	11	77	54	EE	A7	44	FF
1	2C	0D	20	F2	EC	B0	37	E9	42	AB	4D	46	8A	4B	FD	29
2	C7	E2	85	5F	DF	D6	E7	2A	DB	43	63	E1	F1	00	D0	93
3	1B	27	55	25	98	8F	13	8C	17	9D	09	6D	64	57	CD	F3
4	10	05	5A	9C	94	7E	BC	49	BD	D4	AF	C5	50	65	56	0B
5	B5	A1	19	62	59	EA	C1	B4	7C	24	CE	D1	3C	3A	B8	26
6	A9	66	4C	E4	35	32	D5	03	9B	E0	1D	F6	B9	DA	86	48
7	B1	45	39	06	8D	82	D8	E3	CA	CF	AC	51	99	E6	E5	AD
8	22	1A	95	6A	B6	87	3B	97	2B	47	FE	D2	7B	B2	90	F5
9	79	01	36	AA	5D	5C	89	08	3F	6E	C8	33	AE	BE	14	A4
A	69	DD	DC	1C	30	1F	5B	BA	2D	A5	4A	7D	A8	83	6B	F0
B	67	28	D7	72	02	A3	3E	40	7A	BF	EB	6C	73	FC	4E	18
C	CC	F8	53	5E	9A	4F	CB	2F	68	A2	FB	96	34	C2	04	0C
D	F9	DE	C3	7F	38	15	EF	1E	71	D3	B7	C0	0F	21	92	8E
E	61	E8	88	91	70	A0	0E	8B	84	9E	07	60	2E	B3	58	A6
F	0A	41	81	12	C6	6F	3D	78	31	80	52	16	C9	BB	C4	9F

**Table 4:** Structure and format of financial experimental data.

Data Type	Concatenated Core Business Fields	Data Format	128-Bit Block Standardization Rule
Stock trading order data	Stock code + order price + order quantity	Numeric/Structured string	Concatenated fields are ASCII-encoded and padded with 0×00 to 128 bits
Cross-border remittance Data	Payer account + remittance amount + currency	Alphanumeric	Concatenated fields are ASCII-encoded and padded with 0×00 to 128 bits

**Table 5:** Plaintext–ciphertext comparison of financial data blocks.

Data Type	Plaintext (128-Bit, Hexadecimal)	Encrypted Ciphertext (16-Byte, Hexadecimal)	Decryption Result
Stock trading order data	3630A3B3033C631D22E35F83130000000	9A7B2F0E5C4D8A1B6F3E7D9C2A0B58E	Identical
Cross-border remittance data	3632E3F323038A1303031B530C030D055	E3C17A9D0B2F4E8C9A6D3F5B7E0A12C	Identical

**Table 6:** The NBCR and HD performance of the PRNG.

Keys	Altered Keys	NBCR	Correlation Coefficient
IK_1_	Password1	50.01%	0.000971
IK_2_	Password2	50.09%	−0.000891
IK_3_	Password3	49.92%	0.001019
IK_4_	Password4	49.89%	0.002854
IK_5_	Password5	50.11%	−0.000897

**Table 7:** TestU01 results.

Length	Rabbit	Alphabit	BlockAlphabit	Result
2^15^-bit	38/38	17/17	17/17	Passed
2^25^-bit	38/38	17/17	17/17	Passed

**Table 8:** Comparison of encryption algorithms.

Evaluation Indicator	AES	Ref. [25]	Ref. [26]	Ref. [27]	Our Work
Core Structure	Static (10 rounds)	AES-based + modified sine map + blockchain	AES + 1D Logistic Map	AES + temporal redundancy	Full-process dynamic (S-Box/rounds/diffusion/keys)
S-Box Design	1 Static S-Box with weaknesses	1 Static S-Box with weaknesses	dynamic + 3DKGM matrix	1 Static S-Box with weaknesses	2 strong high nonlinearity S-Boxes without weaknesses
Chaotic map	—	1D Sine map	1D Logistic map	—	Non-degenerated 2D hyperchaotic map
Diffusion Operation	Fixed MixColumns matrix	Optimized MixColumns + sine map chaos	3DKGM matrix + chaos-assisted diffusion	Fixed MixColumns matrix	Chaotic-driven dynamic sequence diffusion
Round Key Generation	Fixed mathematical expansion	Blockchain + chaotic sequence	Logistic Map + 3DKGM + XOR	Standard AES + XOR AddRoundKey	Chaos real-time generation (independent keys)
Number of Rounds	Fixed number of rounds	Fixed number of rounds	Fixed number of rounds	Fixed number of rounds	Dynamic number of rounds

## Data Availability

Data is contained within the article.
